# Adaptive mechanical ventilation with automated minimization of mechanical power—a pilot randomized cross-over study

**DOI:** 10.1186/s13054-019-2610-7

**Published:** 2019-10-30

**Authors:** Tobias Becher, Anna Adelmeier, Inéz Frerichs, Norbert Weiler, Dirk Schädler

**Affiliations:** Department of Anesthesiology and Intensive Care Medicine, University Medical Center Schleswig-Holstein, Campus Kiel, Kiel, Germany

**Keywords:** Lung-protective ventilation, Ventilator-induced lung injury, Personalized medicine, Adaptive mechanical ventilation, Mechanical power, Acute respiratory failure

## Abstract

**Background:**

Adaptive mechanical ventilation automatically adjusts respiratory rate (RR) and tidal volume (*V*_T_) to deliver the clinically desired minute ventilation, selecting RR and *V*_T_ based on Otis’ equation on least work of breathing. However, the resulting *V*_T_ may be relatively high, especially in patients with more compliant lungs. Therefore, a new mode of adaptive ventilation (adaptive ventilation mode 2, AVM2) was developed which automatically minimizes inspiratory power with the aim of ensuring lung-protective combinations of *V*_T_ and RR. The aim of this study was to investigate whether AVM2 reduces *V*_T_, mechanical power, and driving pressure (Δ*P*_stat_) and provides similar gas exchange when compared to adaptive mechanical ventilation based on Otis’ equation.

**Methods:**

A prospective randomized cross-over study was performed in 20 critically ill patients on controlled mechanical ventilation, including 10 patients with acute respiratory distress syndrome (ARDS). Each patient underwent 1 h of mechanical ventilation with AVM2 and 1 h of adaptive mechanical ventilation according to Otis’ equation (adaptive ventilation mode, AVM). At the end of each phase, we collected data on *V*_T_, mechanical power, Δ*P*, PaO_2_/FiO_2_ ratio, PaCO_2_, pH, and hemodynamics.

**Results:**

Comparing adaptive mechanical ventilation with AVM2 to the approach based on Otis’ equation (AVM), we found a significant reduction in *V*_T_ both in the whole study population (7.2 ± 0.9 vs. 8.2 ± 0.6 ml/kg, *p* <  0.0001) and in the subgroup of patients with ARDS (6.6 ± 0.8 ml/kg with AVM2 vs. 7.9 ± 0.5 ml/kg with AVM, *p* <  0.0001). Similar reductions were observed for Δ*P*_stat_ (whole study population: 11.5 ± 1.6 cmH_2_O with AVM2 vs. 12.6 ± 2.5 cmH_2_O with AVM, *p* <  0.0001; patients with ARDS: 11.8 ± 1.7 cmH_2_O with AVM2 and 13.3 ± 2.7 cmH_2_O with AVM, *p* = 0.0044) and total mechanical power (16.8 ± 3.9 J/min with AVM2 vs. 18.6 ± 4.6 J/min with AVM, *p* = 0.0024; ARDS: 15.6 ± 3.2 J/min with AVM2 vs. 17.5 ± 4.1 J/min with AVM, *p* = 0.0023). There was a small decrease in PaO_2_/FiO_2_ (270 ± 98 vs. 291 ± 102 mmHg with AVM, *p* = 0.03; ARDS: 194 ± 55 vs. 218 ± 61 with AVM, *p* = 0.008) and no differences in PaCO_2_, pH, and hemodynamics.

**Conclusions:**

Adaptive mechanical ventilation with automated minimization of inspiratory power may lead to more lung-protective ventilator settings when compared with adaptive mechanical ventilation according to Otis’ equation.

**Trial registration:**

The study was registered at the German Clinical Trials Register (DRKS00013540) on December 1, 2017, before including the first patient.

## Background

Adaptive mechanical ventilation provides automated selection and continuous adaptation of basic ventilator parameters like respiratory rate (RR), tidal volume (*V*_T_), and inspiratory time, based on the clinically required minute ventilation (*V̇*_E_) and on the expiratory time constant (RC_e_) of the patient’s respiratory system.

The first adaptive mechanical ventilation mode was introduced in 1994 by Hamilton Medical (Bonaduz, Switzerland) as “adaptive lung ventilation” [1] and was then further developed into the commercially available “adaptive support ventilation” (ASV) [2]. With ASV, the combination of RR and *V*_T_ to achieve the desired *V̇*_E_ is calculated based on Otis’ equation on minimal work of breathing [3]. Several studies have shown that ASV facilitates ventilatory management and shortens the total duration of mechanical ventilation in different patient populations [4–7]. However, it has been observed that ASV may lead to automated delivery of *V*_T_ in excess of what is currently recommended for lung-protective ventilation, especially in patients with more compliant lungs [8].

Recently, adaptive ventilation modes similar to ASV have been released by different manufacturers, including “Adaptive Ventilation Mode” (imtmedical, Buchs, Switzerland), “Work of Breathing Optimized Ventilation” (Salvia Medical, Kronberg, Germany), and “Adaptive Minute Ventilation” (Mindray, Shenzhen, China). All of these select the combination of RR and *V*_T_ according to Otis’ equation and might therefore also deliver large *V*_T_, as observed for ASV by Dongelmans and coworkers [8].

In a recent methodological publication on advanced modes of mechanical ventilation and optimal targeting schemes [9], van der Staay and Chatburn pointed out that Otis’ equation was originally derived to better understand the energetics of unassisted spontaneous breathing, assuming inspiratory muscle pressure to follow a sinusoidal waveform during inspiration. However, all abovementioned adaptive ventilation modes are based on pressure-controlled mechanical ventilation, which delivers a “square-wave” inspiratory pressure during mandatory breaths. Therefore, the authors proposed the concept of “inspiratory power” and derived an equation to select a combination of RR and *V*_T_ that minimizes inspiratory power during adaptive mechanical ventilation, assuming a square-wave pressure pattern as it occurs during pressure-controlled breaths. This concept of inspiratory power assumes that during a pressure-controlled breath, airway pressure rises immediately from positive end-expiratory pressure (PEEP) to the set inspiratory pressure and is maintained stable throughout the course of inspiration. The resulting inspiratory power can then be calculated as follows: first, the inspiratory pressure difference above PEEP is multiplied with *V*_T_ to yield the area of the pressure-volume-loop, which is equal to the work per breath. Subsequently, the work per breath is multiplied with RR to yield the inspiratory power in J/min. The RR that is associated with minimal inspiratory power can then be calculated by the algorithm using a fixed-point iteration (Eq. 2, below).

For a given *V̇*_E_, this should lead to lower *V*_T_ and reduced driving pressure (Δ*P*_stat_) when compared to “traditional” adaptive ventilation based on Otis’ equation [9].

This concept was implemented in a new adaptive ventilation mode (AVM2, imtmedical, Buchs, Switzerland) which was specifically developed to minimize inspiratory power and deliver more “lung-protective” ventilatory settings when compared to adaptive mechanical ventilation based on Otis’ equation.

We hypothesized that, when compared to “traditional” adaptive mechanical ventilation based on Otis’ equation (AVM), ventilator settings selected by AVM2 would be more lung-protective in terms of *V*_T_, Δ*P*, and mechanical power delivered by the ventilator.

## Methods

Between December 2017 and June 2018, we conducted a prospective, randomized, cross-over study including 20 critically ill patients admitted to the interdisciplinary intensive care units (ICUs) of the University Medical Center Schleswig-Holstein, Campus Kiel, Germany. The study was approved by the Ethics Committee of the Medical Faculty of the Christian Albrechts University in Kiel (D551/17) and registered at the German Clinical Trials Register (DRKS00013540) on December 1, 2017, before including the first patient. Written informed consent was obtained from the patient’s legal representatives prior to study inclusion.

### Inclusion criteria

We included intubated adult patients requiring controlled mechanical ventilation, with the presence of an arterial line, which was required for arterial blood gas sampling.

### Exclusion criteria

We excluded patients with significant expiratory flow limitation, as evidenced by an RC_e_ of higher than 1.5 s, patients with a ratio of arterial partial pressure of oxygen to inspired fraction of oxygen (PaO_2_/FiO_2_) of less than 100 mmHg, arterial pH of less than 7.2, or arterial partial pressure of carbon dioxide (PaCO_2_) of more than 70 mmHg despite optimization of mechanical ventilation, severe hemodynamic instability, high-frequency oscillatory ventilation, and spontaneous breathing activity.

### Study procedure

**Fig. 1:**
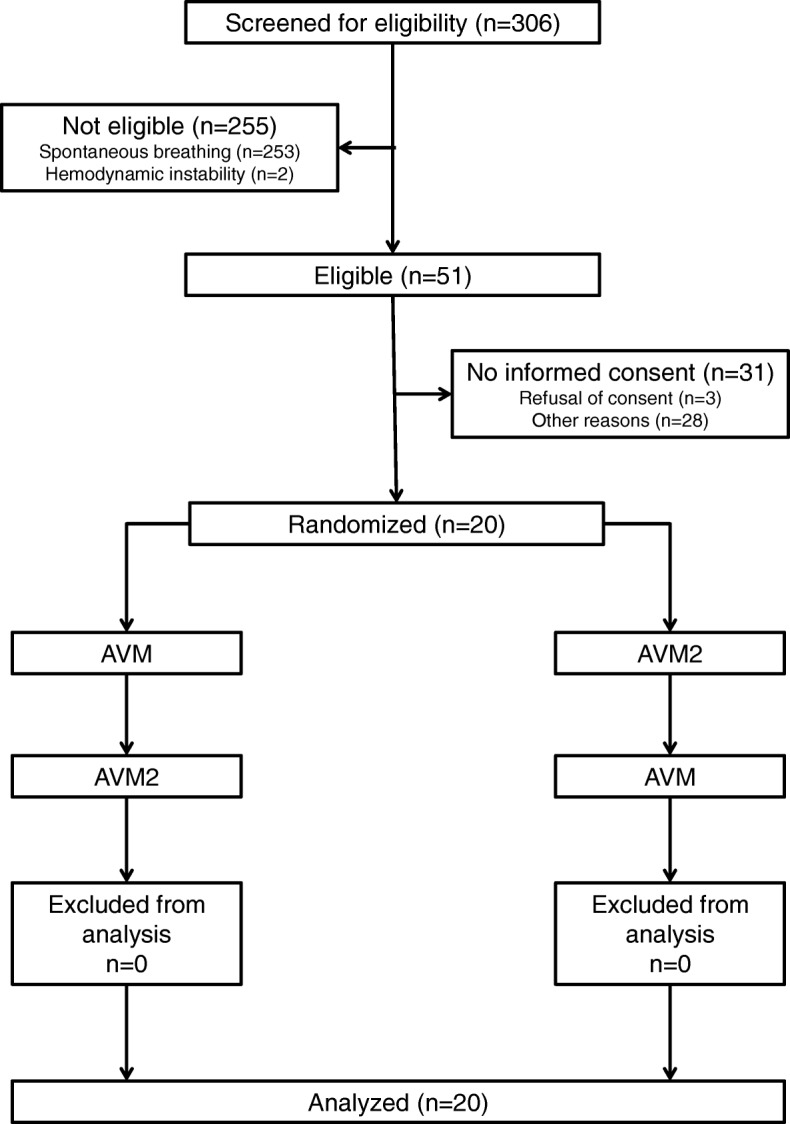
CONSORT diagram. AVM, adaptive ventilation mode with selection of respiratory rate and tidal volume according to Otis’ equation; AVM2, adaptive ventilation mode with selection of respiratory rate and tidal volume to minimize inspiratory power

Before study inclusion, all patients were ventilated in a conventional pressure-controlled mode. After obtaining written informed consent, patients were randomized in a 1:1 ratio to one of two groups: group “AVM-AVM2”, first ventilated according to Otis’ equation with AVM and then according to “minimized inspiratory power” with AVM2, or group “AVM2-AVM”, ventilated with both modes in the reversed order (Fig. 1). Randomization was performed by randomly selecting and opening one of 20 sealed envelopes for every patient. Before randomization, it was verified that the last clinically selected *V̇*_E_ was adequate to deliver clinically acceptable pH and gas exchange. If any changes in *V̇*_E_ were necessary to achieve the desired pH of more than 7.25 and the desired range of PaCO_2_ (35 mmHg < PaCO_2_ < 70 mmHg), the ventilator settings were adjusted by the attending ICU physician according to local clinical practice.

**Fig. 2:**
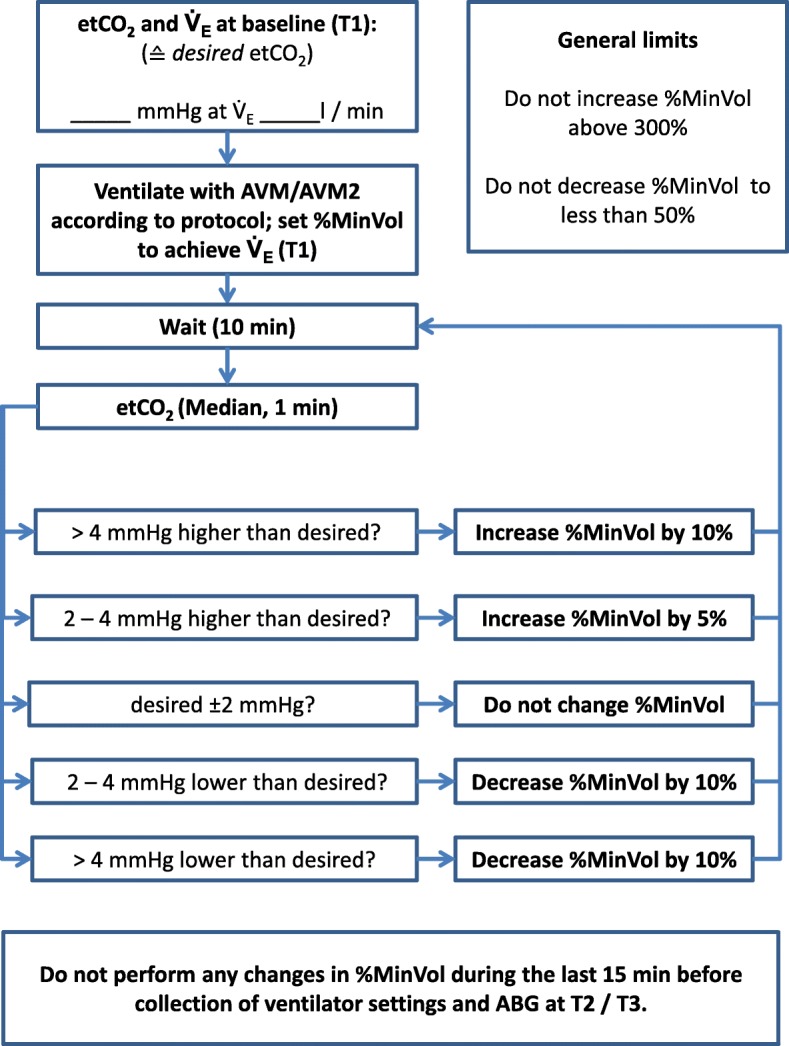
Adjustment of target minute ventilation (%MinVol) during ventilation with either mode. End-tidal partial pressure of carbon dioxide (etCO_2_) and current minute ventilation (*V̇*_E_) during stable baseline conditions were documented. During ventilation with either mode, %MinVol was adjusted according to the protocol in order to keep etCO_2_ within a range of baseline etCO_2_ ± 2 mmHg. AVM, adaptive ventilation mode with selection of respiratory rate and tidal volume according to Otis’ equation; AVM2, adaptive ventilation mode with selection of respiratory rate and tidal volume to minimize inspiratory power; ABG, arterial blood gas analysis

After randomization, the last clinically selected *V̇*_E_ during conventional ventilation was chosen as the target *V̇*_E_ for the first adaptive mode to be investigated and end-tidal partial pressure of carbon dioxide (etCO_2_) was documented and selected as “desired” target value. Depending on group allocation, patients were then switched to either AVM or AVM2 and ventilated with the selected mode for the duration of 1 h. If necessary, target *V̇*_E_ was adjusted to keep etCO_2_ constant (Fig. 2). After 1 h, patients previously ventilated with AVM were switched to AVM2 and vice versa.

With AVM, the RR necessary to achieve target *V̇*_E_ was automatically calculated by the ventilator according to a modification of Otis’ equation under the assumption of an anatomical dead space (VD_A_) of 2.2 ml/kg predicted body weight:

1 $$ \mathrm{RR}=\frac{\sqrt{1+\frac{4\cdot {\pi}^2\cdot {\mathrm{RC}}_{\mathrm{e}}\cdot \left({\dot{V}}_{\mathrm{E}}-{\mathrm{VD}}_{\mathrm{A}}\cdot \mathrm{RR}\right)}{{\mathrm{VD}}_{\mathrm{A}}}\hbox{-} 1}}{2\cdot {\pi}^2\cdot {\mathrm{RC}}_{\mathrm{e}}} $$

With AVM2, the RR to achieve target *V̇*_E_ was calculated by the ventilator according to the following equation:

2 $$ \mathrm{RR}=\frac{{\dot{V}}_{\mathrm{E}}}{2\cdot {\mathrm{VD}}_{\mathrm{A}}}\cdot \left(1\hbox{-} \frac{1}{2\cdot \mathrm{RR}\cdot {\mathrm{RC}}_{\mathrm{e}}\left(\frac{1}{e^2\cdot \mathrm{RR}\cdot {\mathrm{RC}}_{\mathrm{e}}}-1\right)}\right) $$

(RR = respiratory rate; *π* = Pi ≈ 3.14; *e* = Euler’s number ≈ 2.72; RC_e_ = expiratory time constant; *V̇*_E_ = expiratory minute ventilation; VD_A_ = anatomical dead space)

The equations for AVM and AVM2 are automatically solved for RR by the ventilator using a fixed-point iteration [9].

Clinically selected PEEP was maintained unchanged throughout the whole study period.

### Study endpoints

The primary endpoint of the study was the automatically delivered *V*_T_ in ml/kg predicted body weight. Secondary endpoints were Δ*P*_stat_ in cmH_2_O, mechanical power in J/min, mean airway pressure (*P*_aw,mean_) in cmH_2_O, PaO_2_/FiO_2_, PaCO_2_, pH, alveolar minute ventilation (*V̇*_A_), mean arterial pressure (MAP), and heart rate (HR).

For assessment of the study endpoints, ventilator data on *V*_T_, Δ*P*, mechanical power, and *P*_aw,mean_ as well as hemodynamic data on MAP and HR were averaged during the last 5 min of each 1-h period. Real-time data of flow and airway pressure were stored by the ventilator at a sampling rate of 100 Hz and analyzed off-line after the end of the study procedure. An arterial blood gas (ABG) sample was taken to assess PaO_2_/FiO_2_, PaCO_2_, and pH. Δ*P* was calculated as the difference between end-inspiratory plateau pressure during an inspiratory hold maneuver of 2–3 s and PEEP. Static respiratory system compliance (*C*_rs_) was then calculated by dividing expired *V*_T_ by Δ*P*_stat_.

Total mechanical power per breath was derived from the integral of the inspiratory pressure-volume curve for each breath in the 5-min interval and multiplied with RR to obtain a result in Joules per minute (J/min).

To assess *V̇*_A_, physiological dead space (VD) was estimated according to the modified Bohr equation [10]. Subsequently, VD was subtracted from *V*_T_ to calculate alveolar *V*_T_. Finally, alveolar *V*_T_ was multiplied with RR to yield *V̇*_A_.

### Statistical analysis

A sample size of *n* = 20 was calculated to detect an average difference in *V*_T_ of 1 ml/kg assuming a standard deviation of change in *V*_T_ of 1.5 ml/kg with a power of 80% at a significance level of *p* ≤ 0.05. Statistical analyses were performed with GraphPad Prism 5.0 (GraphPad Software, La Jolla, USA). Data were assessed for normal distribution using the D’Agostino&Pearson omnibus K2 normality test. Parametric continuous data are expressed as mean ± SD, whereas nonparametric continuous data are expressed as median (interquartile range). Comparisons between AVM and AVM2 were performed using the paired two-sided *t* test for parametric data and the Wilcoxon matched-pairs test for nonparametric data. The relationship between automatically selected *V*_T_ and *C*_rs_ was assessed with Pearson’s correlation coefficient (*r*) for both modes.

## Results

Details on numbers of patients screened, excluded, randomized, and analyzed are shown in the CONSORT-diagram (Fig. 1). Basic patient characteristics are summarized in Table 1. According to the Berlin definition [11], ten patients had acute respiratory distress syndrome (ARDS), including five cases of mild ARDS and five cases of moderate ARDS.

**Table 1:** Patient characteristics at study inclusion

Gender male/female (*n*)	13/7
Age (years)	64 ± 11
Height (cm)	181 ± 9
Actual body weight (kg)	82 ± 13
Predicted body weight (kg)	74 ± 10
Duration of MV before study inclusion (days)	5 ± 3
PaO_2_/FiO_2_ (mmHg)	258 ± 91
PaCO_2_ (mmHg)	43 ± 6
*C*_rs_ (ml/cmH_2_O)	55 ± 13
PEEP (cmH_2_O)	8 (8–10)
*V*_T_ (ml/kg predicted body weight)	7.5 ± 0.8
RR (1/min)	15.3 ± 1.8

Parametric data are presented as mean ± standard deviation; nonparametric data are presented as median (interquartile range). *MV* mechanical ventilation, *PaO*_*2*_*/FiO*_*2*_ ratio of arterial partial pressure of oxygen to inspired fraction of oxygen, *PaCO*_*2*_ arterial partial pressure of carbon dioxide, *C*_*rs*_ static respiratory system compliance, *PEEP* positive end-expiratory pressure, *V*_*T*_ tidal volume, *RR* respiratory rate

The numerical results for the whole study population are presented in Table 2:

**Table 2:** Results

Parameter	AVM	AVM2	*p* value
*V*_T_ (ml/kg)	8.2 ± 0.6	7.2 ± 0.9	< 0.0001
Δ*P*_stat_ (cmH_2_O)	12.6 ± 2.5	11.5 ± 1.6	0.0022
*P*_insp_ (cmH_2_O)	23.9 ± 3.5	20.7 ± 2.8	< 0.0001
RR (1/min)	12.9 ± 1.7	15.8 ± 2.6	< 0.0001
Mechanical Power (J/min)	18.6 ± 4.6	16.8 ± 3.9	0.0024
*V̇*_A_	4.5 ± 0.9	4.6 ± 1.0	0.71
*P*_aw,mean_ (cmH_2_O)	14.0 (12.9–14.6)	14.6 (13.6–16.1)	0.0008
*C*_rs_ (ml/cmH_2_O)	51.8 ± 12.1	47.7 ± 12.2	0.0043
*R*_insp_ (cmH_2_O/l/s)	11.1 ± 2.3	10.0 ± 1.7	0.0004
RC_e_ (s)	0.82 ± 0.22	0.83 ± 0.26	0.68
PaO_2_/FiO_2_ (mmHg)	291 ± 102	270 ± 98	0.03
PaCO_2_ (mmHg)	38.4 ± 4.2	39.1 ± 5.8	0.33
pH	7.46 ± 0.07	7.46 ± 0.07	0.37
MAP (mmHg)	84 ± 12	83 ± 12	0.87
HR (1/min)	70 ± 18	71 ± 18	0.42

Parametric data are presented as mean ± standard deviation; nonparametric data are presented as median (interquartile range). *p* values were calculated using a two-sided paired *t* test or a Wilcoxon matched-pairs test for parametric and nonparametric data, respectively. *V*_*T*_ tidal volume, *ΔP*_*stat*_ driving pressure (measured during end-inspiratory occlusion maneuver), *P*_*insp*_ inspiratory airway pressure (measured during ongoing ventilation), *RR* respiratory rate, *V̇*_*A*_ alveolar minute ventilation, *P*_*aw,mean*_ mean airway pressure, *C*_*rs*_ static respiratory system compliance, *R*_*insp*_ inspiratory resistance, *RC*_*e*_ expiratory time constant, *PaO*_*2*_*/FiO*_*2*_ ratio of arterial partial pressure of oxygen to inspired fraction of oxygen, *PaCO*_*2*_ arterial partial pressure of carbon dioxide, *MAP* mean arterial pressure, *HR* heart rate

In comparison to AVM, ventilation with AVM2 led to statistically significant reductions in *V*_T_ by 1.05 ± 0.4 ml/kg, Δ*P*_stat_ by 1.1 ± 1.3 cmH_2_O, and total mechanical power by 1.8 ± 2.3 J/min. RR and *P*_aw,mean_ were significantly higher with AVM2. *V̇*_A_ did not differ between the two modes. Inspiration-to-expiration ratio (I:E) was 1:2.0 ± 0.5 with AVM and 1:1.1 ± 0.1 with AVM2, resulting in a significantly higher *P*_aw,mean_ with AVM2 despite lower *V*_T_ and ΔP_stat_.

In ABG samples, we found a small but statistically significant reduction in PaO_2_/FiO_2_ by 21 ± 39 mmHg with AVM2 and no significant differences in PaCO_2_ or pH. There were no differences in hemodynamics between both modes. We observed a statistically significant reduction in static *C*_rs_ by 4.1 ± 5.6 ml/cmH_2_O with AVM2 as compared to AVM.

In the ten patients with ARDS, *V*_T_ was reduced by 1.3 ± 0.3 ml/kg with AVM2 (6.6 ± 0.8 ml/kg as opposed to 7.9 ± 0.5 ml/kg with AVM, *p* <  0.0001). In this subgroup, Δ*P*_stat_ was reduced by 1.5 ± 1.2 cmH_2_O (11.8 ± 1.7 cmH_2_O and 13.3 ± 2.7 cmH_2_O with AVM2 and AVM, respectively; *p* = 0.0044) and total mechanical power was reduced by 1.9 ± 1.6 J/min to a value of 15.6 ± 3.2 J/min with AVM2 (17.5 ± 4.1 J/min with AVM, *p* = 0.006). The complete results of the subgroup of patients with ARDS are presented in Table 3:

**Table 3:** Results for subgroup of patients with acute respiratory distress syndrome (ARDS)

Parameter	AVM	AVM2	*p* value
*V*_T_ (ml/kg)	7.9 ± 0.5	6.6 ± 0.8	< 0.0001
Δ*P*_stat_ (cmH_2_O)	13.3 ± 2.7	11.8 ± 1.7	0.0044
*P*_insp_ (cmH_2_O)	23.0 ± 2.6	20.5 ± 2.0	< 0.0001
RR (1/min)	13.0 ± 2.0	16.3 ± 2.9	0.0001
Mechanical Power (J/min)	17.5 ± 4.6	15.6 ± 3.2	0.006
*V̇*_A_	4.1 ± 0.7	3.9 ± 0.9	0.26
*P*_aw, mean_ (cmH_2_O)	13.7 ± 0.9	14.3 ± 1.1	0.11
*C*_rs_ (ml/cmH_2_O)	47.3 ± 9.1	43.8 ± 11.9	0.12
*R*_insp_ (cmH_2_O/l/s)	10.8 ± 2.7	9.5 ± 1.6	0.02
RC_e_ (s)	0.73 ± 0.18	0.71 ± 0.23	0.53
PaO_2_/FiO_2_ (mmHg)	218 ± 61	195 ± 55	0.01
PaCO_2_ (mmHg)	38.5 ± 4.0	40.2 ± 6.1	0.19
pH	7.44 ± 0.08	7.43 ± 0.08	0.18
MAP (mmHg)	82 ± 12	81 ± 12	0.75
HR (1/min)	63 ± 11	64 ± 11	0.42

Parametric data are presented as mean ± standard deviation; nonparametric data are presented as median (interquartile range). *p* values were calculated using a two-sided paired *t* test or a Wilcoxon matched-pairs test for parametric and nonparametric data, respectively. *V*_*T*_ tidal volume, *ΔP*_*stat*_ driving pressure (measured during end-inspiratory occlusion maneuver), *P*_*insp*_ inspiratory airway pressure (measured during ongoing ventilation), *RR* respiratory rate, *V̇*_*A*_ alveolar minute ventilation, *P*_*aw,mean*_ mean airway pressure, *C*_*rs*_ static respiratory system compliance, *R*_*insp*_ inspiratory resistance, *RC*_*e*_ expiratory time constant, *PaO*_*2*_*/FiO*_*2*_ ratio of arterial partial pressure of oxygen to inspired fraction of oxygen, *PaCO*_*2*_ arterial partial pressure of carbon dioxide, *MAP* mean arterial pressure, *HR* heart rate

**Fig. 3:**
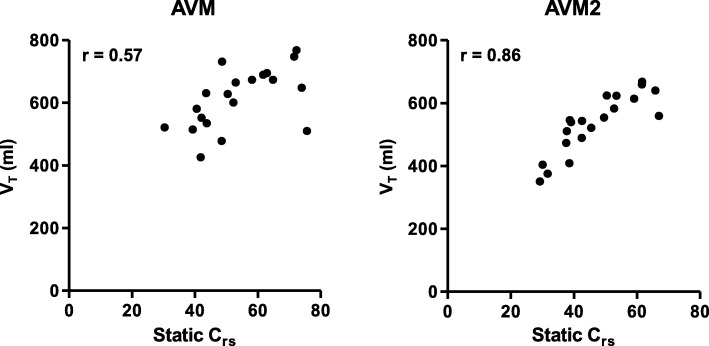
Correlation between tidal volume (*V*_T_) and static respiratory system compliance (*C*_rs_) for the two different modes Pearson’s *r* = 0.57 (*p* = 0.0094) for AVM and 0.86 (*p* < 0.0001) for AVM2. AVM, adaptive ventilation mode with selection of respiratory rate and tidal volume according to Otis’ equation; AVM2, adaptive ventilation mode with selection of respiratory rate and tidal volume to minimize inspiratory power

Both with AVM and with AVM2, *V*_T_ was positively correlated to static *C*_rs_ (*r* = 0.86, *p* < 0.0001 for AVM2 and *r* = 0.57, *p* = 0.009 for AVM; Fig. 3). Additional data on the individual correlations between *V*_T_, Δ*P*_stat_, and MP with RC_e_ for both modes and the differences between modes are presented in Additional file 1.

## Discussion

In this randomized cross-over study, we found that adaptive mechanical ventilation with AVM2, selecting RR and *V*_T_ to minimize inspiratory power, led to significant reductions in *V*_T_, Δ*P*, and mechanical power when compared to adaptive mechanical ventilation according to Otis’ equation while providing similar alveolar ventilation.

Several studies have demonstrated the lung-protective properties of lowering *V*_T_ in patients with ARDS [12–14]. In patients without ARDS, lower *V*_T_ has been suggested to be protective [15, 16], but a recent study comparing low vs. intermediate *V*_T_ failed to show any benefit in this patient population [17]. Amato et al. provided data supporting the hypothesis that Δ*P* may be more important than *V*_T_ for preventing death in patients with ARDS [18].

In clinical practice, both *V*_T_ and Δ*P* can be reduced by ventilating patients with an increased RR. However, a recent animal study demonstrated that increasing RR may, by itself, lead to lung damage when the total mechanical power delivered to the lung exceeds a certain threshold [19]. A retrospective analysis of more than 8000 ICU patient data sets revealed that high mechanical power was independently associated with increased mortality and prolonged duration of mechanical ventilation [20].

Therefore, our findings of lower *V*_T_, Δ*P*, and mechanical power suggest that with AVM2, the automatically selected ventilator settings were more lung-protective than those selected according to Otis’ equation.

On the other hand, despite higher *P*_aw,mean_, we observed a small but statistically significant reduction in PaO_2_/FiO_2_ and static *C*_rs_ with AVM2. This is in line with previous findings of higher *V*_T_ being associated with more aerated lung tissue at end-expiration and increased cyclic recruitment-derecruitment [21, 22]. Indeed, in the landmark ARDS Network trial comparing lung-protective *V*_T_ of 6 ml per kg with “traditional” *V*_T_ of 12 ml per kg, the patients randomized to lung-protective ventilation had lower PaO_2_/FiO_2_ on days 1 and 3, despite similar *P*_aw,mean_ in both groups [13].

In most situations, a reduction in PaO_2_/FiO_2_ by 20 mmHg, as observed in our study, may not be clinically relevant. However, in severely hypoxemic patients, even a small deterioration in oxygenation can become a clinical problem. In these cases, one might attempt to counteract this by increasing PEEP, which could, however, lead to an increase in total mechanical power [23], or by performing short intermittent recruitment maneuvers (“sighs”) [24].

Despite minimization of inspiratory power, the average *V*_T_ of 7.2 ± 0.9 ml/kg with AVM2 was still slightly higher than the value of 6 ml/kg generally recommended for patients with ARDS. However, in the subgroup of patients with ARDS, AVM2 selected an average *V*_T_ of 6.6 ± 0.8 ml/kg. Moreover, the average Δ*P* selected by AVM2 was small, and no patient was ventilated with Δ*P* exceeding 15 cmH_2_O with the new mode. As the result of normalizing *V*_T_ to *C*_rs_, Δ*P* is proportional to *V*_T_ normalized to the functional size of the lung [18]. On average, the patients included in our study had only slightly impaired *C*_rs_. Our analysis of the correlation between static *C*_rs_ and *V*_T_ revealed that with AVM2, *V*_T_ was strongly correlated to *C*_rs_ (*r* = 0.86, *p* < 0.0001; Fig. 3, right panel). Therefore, it appears that adaptive mechanical ventilation with minimization of inspiratory power leads to an automated scaling of *V*_T_ to functional lung size, an effect that was less pronounced with adaptive mechanical ventilation according to Otis’ equation (Fig. 3, left panel).

Our study has several limitations. As a pilot randomized cross-over study, it did not assess any long-term effects of mechanical ventilation with automated minimization of inspiratory power on clinical outcomes. Moreover, we excluded a rather large proportion of patients for “presence of spontaneous breathing activity”. We decided to do this because at the time we performed the study, the algorithm of AVM2 chose the “traditional” approach of selecting RR and *V*_T_ according to Otis’ equation whenever a patient-triggered breath was detected. As the concept of “minimal inspiratory power” assumes a square-wave pressure pattern during inspiration as opposed to the sinusoidal pressure pattern assumed by Otis’ equation [9], it was a logical approach to apply this concept only to pressure-controlled mandatory ventilation in a first step. However, in the meantime, the algorithm of AVM2 for patients with spontaneous respiratory activity has been further optimized to prevent excessive *V*_T_ and inspiratory pressure support. A future study should investigate the improved algorithm in patients triggering the ventilator.

Another limitation is that we did not directly measure transpulmonary mechanical power applied to the lungs, as this would have required insertion of an esophageal balloon catheter and recording of transpulmonary pressure. Instead, we assessed mechanical power delivered to the respiratory system as a whole, with an unknown proportion of power distending the patient’s chest wall. With the randomized cross-over design of our study, we sought to minimize any bias resulting from this limitation. In the recently published study by Serpa Neto et al. on mechanical power and mortality in critically ill patients [20], mechanical power was also derived from global ventilator parameters with no data on transpulmonary pressure available. Therefore, it appears that despite the valid assumption that mechanical power assessed by direct measurements of transpulmonary pressure may be a more precise “biomarker” of potential damage to the lungs [25], mechanical power delivered to the respiratory system as a whole is still associated with clinically relevant changes in patient outcomes.

Recently, the original ASV (Hamilton Medical) has also been modified to reduce the delivered *V*_T_ and driving pressure (ASV 1.1). To our knowledge, there are no controlled studies comparing ASV 1.1 to ASV based on Otis’ equation or to AVM. It would be interesting to assess the differences between ASV 1.1 and AVM2 in a future study.

We must admit that there is currently no evidence of improved long-term outcomes when using adaptive mechanical ventilation in the management of critically ill patients with and without ARDS. Observational studies have repeatedly shown that the implementation of lung-protective ventilation in daily clinical practice is slow and incomplete. In the LUNG SAFE study, less than two thirds of patients with ARDS were ventilated with tidal volumes of 8 or less ml/kg predicted body weight and there was no evidence to suggest that lower tidal volumes were used in patients with a less compliant respiratory system [26]. Conceivably, a broader application of adaptive mechanical ventilation modes optimized for lung-protective ventilation might improve the management of mechanically ventilated patients in daily clinical practice.

## Conclusions

In conclusion, our results indicate that adaptive mechanical ventilation with minimization of inspiratory power may be more lung-protective in patients undergoing controlled mechanical ventilation without spontaneous efforts than adaptive mechanical ventilation according to Otis’ equation.

## Supplementary information

**Additional file 1.** Plots of V_T_, ΔP_stat_ and mechanical power against RC_e_. This additional file presents data on the correlation between V_T_, ΔP_stat_ and mechanical power with RC_e_ for AVM and AVM2 as well as the difference of these parameters between AVM and AVM2 plotted against RC_e_.

## Data Availability

The data are available from the corresponding author on reasonable request.

## Abbreviations

ABG: Arterial blood gas
ARDS: Acute respiratory distress syndrome
AVM: Adaptive ventilation mode
AVM2: Adaptive ventilation mode 2
*C*_rs_: Respiratory system compliance
Δ*P*_stat_: Driving pressure
etCO_2_: End-tidal carbon dioxide
FiO_2_: Inspired fraction of oxygen
HR: Heart rate
ICU: Intensive care unit
PaCO_2_: Arterial partial pressure of carbon dioxide
PaO_2_: Arterial partial pressure of oxygen
PEEP: Positive end-expiratory pressure
*P*_insp_: Inspiratory airway pressure
MAP: Mean arterial pressure
*P*_aw,mean_: Mean airway pressure
RC_e_: Expiratory time constant
RR: Respiratory rate
*V*_T_: Tidal volume
*V̇*_A_: Alveolar minute ventilation
*V̇*_E_: Expiratory minute ventilation

## References

[1] Laubscher TP, Heinrichs W, Weiler N, Hartmann G, Brunner JX (1994). An adaptive lung ventilation controller. IEEE Trans Biomed Eng.

[2] Brunner JX, Iotti GA (2002). Adaptive support ventilation (ASV). Minerva Anestesiol.

[3] Otis AB, Fenn WO, Rahn H (1950). Mechanics of breathing in man. J Appl Physiol.

[4] Gruber PC, Gomersall CD, Leung P, Joynt GM, Ng SK, Ho KM, Underwood MJ (2008). Randomized controlled trial comparing adaptive-support ventilation with pressure-regulated volume-controlled ventilation with automode in weaning patients after cardiac surgery. Anesthesiology.

[5] Kirakli C, Ozdemir I, Ucar ZZ, Cimen P, Kepil S, Ozkan SA (2011). Adaptive support ventilation for faster weaning in COPD: a randomised controlled trial. Eur Respir J.

[6] Kirakli C, Naz I, Ediboglu O, Tatar D, Budak A, Tellioglu E (2015). A randomized controlled trial comparing the ventilation duration between adaptive support ventilation and pressure assist/control ventilation in medical patients in the ICU. Chest.

[7] Zhu F, Gomersall CD, Ng SK, Underwood MJ, Lee A (2015). A randomized controlled trial of adaptive support ventilation mode to wean patients after fast-track cardiac valvular surgery. Anesthesiology.

[8] Dongelmans DA, Paulus F, Veelo DP, Binnekade JM, Vroom MB, Schultz MJ (2011). Adaptive support ventilation may deliver unwanted respiratory rate–tidal volume combinations in patients with acute lung injury ventilated according to an open lung concept. Anesthesiology.

[9] Van der Staay M, Chatburn RL (2018). Advanced modes of mechanical ventilation and optimal targeting schemes. Intensive Care Med Exp.

[10] Lucangelo U, Bernabè F, Vatua S, Degrassi G, Villagrà A, Fernandez R, Romero PV, Saura P, Borelli M, Blanch L (2008). Prognostic value of different dead space indices in mechanically ventilated patients with acute lung injury and ARDS. Chest.

[11] ARDS Definition Task Force, Ranieri VM, Rubenfeld GD, Thompson BT, Ferguson ND, Caldwell E, Fan E, Camporota L, Slutsky AS: Acute respiratory distress syndrome: the Berlin Definition. JAMA 2012; 307:2526–2533. 10.1001/jama.2012.5669 22797452

[12] Amato MB, Barbas CS, Medeiros DM, Magaldi RB, Schettino GP, Lorenzi-Filho G, Kairalla RA, Deheinzelin D, Munoz C, Oliveira R, Takagaki TY, Carvalho CR (1998). Effect of a protective-ventilation strategy on mortality in the acute respiratory distress syndrome. N Engl J Med.

[13] Brower RG, Matthay MA, Morris A, Schoenfeld D, Thompson BT, Wheeler A, The Acute Respiratory Distress Syndrome Network (2000). Ventilation with lower tidal volumes as compared to traditional tidal volumes for acute lung injury and the acute respiratory distress syndrome. N Engl J Med.

[14] Villar JM, Kacmarek RM, Pérez-Méndez L, Aguiree-Jaime A, for the ARIES Network (2006). A high positive end-expiratory pressure, low tidal volume ventilatory strategy improves outcome in persistent acute respiratory distress syndrome: a randomized, controlled trial. Crit Care Med.

[15] Determann RM, Royakkers A, Wolthuis EK, Vlaar AP, Choi G, Paulus F, Hofstra JJ, de Graaff MJ, Korevaar JC, Schultz MJ (2010). Ventilation with lower tidal volumes as compared with conventional tidal volumes for patients without acute lung injury: a preventive randomized controlled trial. Crit Care.

[16] Serpa Neto A, Simonis FD, Barbas CS, Biehl M, Determann RM, Elmer J, Friedman G, Gajic O, Goldstein JN, Horn J, Juffermans NP, Linko R, de Oliveira RP, Sundar S, Talmor D, Wolthuis EK, de Abreu MG, Pelosi P, Schultz MJ (2014). Association between tidal volume size, duration of ventilation, and sedation needs in patients without acute respiratory distress syndrome: an individual patient data meta-analysis. Intensive Care Med.

[17] Simonis FD, Serpa Neto A, Binnekade JM, Braber A, KCM B, Determann RM, Goekoop GJ, Heidt J, Horn J, Innemee G, de Jonge E, Juffermans NP, Spronk PE, Steuten LM, Tuinman PR, de Wilde RBP, Vriends M, Gama de Abreu M, Pelosi P, Schultz MJ, Writing Group for the PReVENT Investigators (2018). Effect of a low vs intermediate tidal volume strategy on ventilator-free days in intensive care unit patients without ARDS: a randomized clinical trial. JAMA.

[18] Amato MB, Meade MO, Slutsky AS, Brochard L, Costa EL, Schoenfeld DA, Stewart TE, Briel M, Talmor D, Mercat A, Richard JC, Carvalho CR, Brower RG (2015). Driving pressure and survival in the acute respiratory distress syndrome. N Engl J Med.

[19] Cressoni M, Gotti M, Chiurazzi C, Massari D, Algieri I, Amini M, Cammaroto A, Brioni M, Montaruli C, Nikolla K, Guanziroli M, Dondossola D, Gatti S, Valerio V, Vergani GL, Pugni P, Cadringher P, Gagliano N, Gattinoni L (2016). Mechanical power and development of ventilator-induced lung injury. Anesthesiology.

[20] Serpa Neto A, Deliberato RO, Johnson AEW, Bos LD, Amorim P, Pereira SM, Cazati DC, Cordioli RL, Correa TD, Pollard TJ, Schettino GPP, Timenetsky KT, Celi LA, Pelosi P, Gama de Abreu M, Schultz MJ; PROVE Network Investigators: Mechanical power of ventilation is associated with mortality in critically ill patients: an analysis of patients in two observational cohorts. Intensive Care Med 2018; 44:1914–1922. 10.1007/s00134-018-5375-6 30291378

[21] Bruhn A, Bugedo D, Riquelme F, Varas J, Retamal J, Besa C, Cabrera C, Bugedo G (2011). Tidal volume is a major determinant of cyclic recruitment-derecruitment in acute respiratory distress syndrome. Minerva Anestesiol.

[22] Retamal J, Libuy J, Jiménez M, Delgado M, Besa C, Bugedo G, Bruhn A (2013). Preliminary study of ventilation with 4 ml/kg tidal volume in acute respiratory distress syndrome: feasibility and effects on cyclic recruitment - derecruitment and hyperinflation. Crit Care.

[23] Collino F, Rapetti F, Vasques F, Maiolo G, Tonetti T, Romitti F, Niewenhuys J, Behnemann T, Camporota L, Hahn G, Reupke V, Holke K, Herrmann P, Duscio E, Cipulli F, Moerer O, Marini JJ, Quintel M, Gattinoni L (2019). Positive end-expiratory pressure and mechanical power. Anesthesiology.

[24] Constantin JM, Godet T, Jabaudon M, Bazin JE, Futier E. Recruitment maneuvers in acute respiratory distress syndrome. Ann Transl Med. 2017;5(14):290. 10.21037/atm.2017.07.09. 10.21037/atm.2017.07.09 PMC5537118 28828365

[25] Brochard L, Bersten A (2019). Mechanical power – a biomarker for the lung?. Anesthesiology.

[26] Bellani G, Laffey JG, Pham T, Fan E, Brochard L, Esteban A, Gattinoni L, van Haren F, Larsson A, McAuley DF, Ranieri M, Rubenfeld G, Thompson BT, Wrigge H, Slutsky AS, Pesenti A; LUNG SAFE Investigators; ESICM Trials Group. Epidemiology, patterns of care, and mortality for patients with acute respiratory distress syndrome in intensive care units in 50 countries. JAMA. 2016;315(8):788–800. doi: 10.1001/jama.2016.0291. 10.1001/jama.2016.0291 26903337

