# Supplementary material for: Adaptive mechanical ventilation with automated minimization of mechanical power—a pilot randomized cross-over study
Source: Crit Care. 2019 Oct 30;23:338. doi: 10.1186/s13054-019-2610-7 (PMC6822420; doi:10.1186/s13054-019-2610-7)
Supplement: Supplementary file 1 — Additional file 1. Plots of VT, ΔPstat and mechanical power against RCe. This additional file presents data on the correlation between VT, ΔPstat and mechanical power with RCe for AVM and AVM2 as well as the difference of these parameters between AVM and AVM2 plotted against RCe. [file 13054_2019_2610_MOESM1_ESM.pdf]

# Adaptive mechanical ventilation with automated minimization of mechanical power – a pilot randomized cross-over study

Tobias Becher, M.D.; Anna Adelmeier, M.D., Inéz Frerichs, M.D., Ph.D., Norbert Weiler, M.D., Dirk Schädler, M.D.; Department of Anesthesiology and Intensive Care Medicine, University Medical Center Schleswig-Holstein, Campus Kiel.

**Corresponding Author:** Tobias Becher, Department of Anesthesiology and Intensive Care Medicine, University Medical Center Schleswig-Holstein, Campus Kiel; +49 431 500 20980; tobias.becher@uksh.de

# - Additional File -

In this additional file, we present data on the correlation between tidal volume ($V_T$), driving pressure ($\Delta P_{stat}$), mechanical power (MP) and the expiratory time constant ($RC_e$) during adaptive mechanical ventilation with selection of respiratory rate (RR) and $V_T$ according to Otis' equation on least work of breathing (adaptive ventilation mode, AVM) and with automated minimization of inspiratory power (adaptive ventilation mode 2, AVM2) to highlight the influence of the dynamics of expiration on the automated optimization of mechanical ventilation with both modes.

We show a positive correlation beween $V_T$ and $RC_e$ for both modes, which is more pronounced for AVM2 (Figure S1, left panel of the top and middle rows). As $RC_e$ can be approximated by multiplying resistance with respiratory system compliance ($C_{rs}$), this is in line with our finding of a stronger correlation between $V_T$ and $C_{rs}$ with AVM2 when compared to AVM (main manuscript Figure 3).

There was no correlation between $\Delta P_{stat}$ or MP and $RC_e$ both for AVM and AVM2 (Figure S1, middle and right panels of the top and middle rows).

When plotting the difference in $V_T$, $\Delta P_{stat}$ or MP between AVM and AVM2 against the average $RC_e$ for both modes (Figure S1, bottom row), we found no significant correlation between the differences in these parameters and $Rc_e$. Nevertheless, it appears that there might be a trend towards a stronger reduction in MP with AVM2 in patients with higher $RC_e$ when removing one outlier who had a severe decrease in respiratory system compliance ($C_{rs}$) during ventilation with AVM2. This decrease in $C_{rs}$ caused an increase in MP and $\Delta P_{stat}$ despite ventilation with lower $V_T$ (Figure S1, middle and right panels of the bottom row).

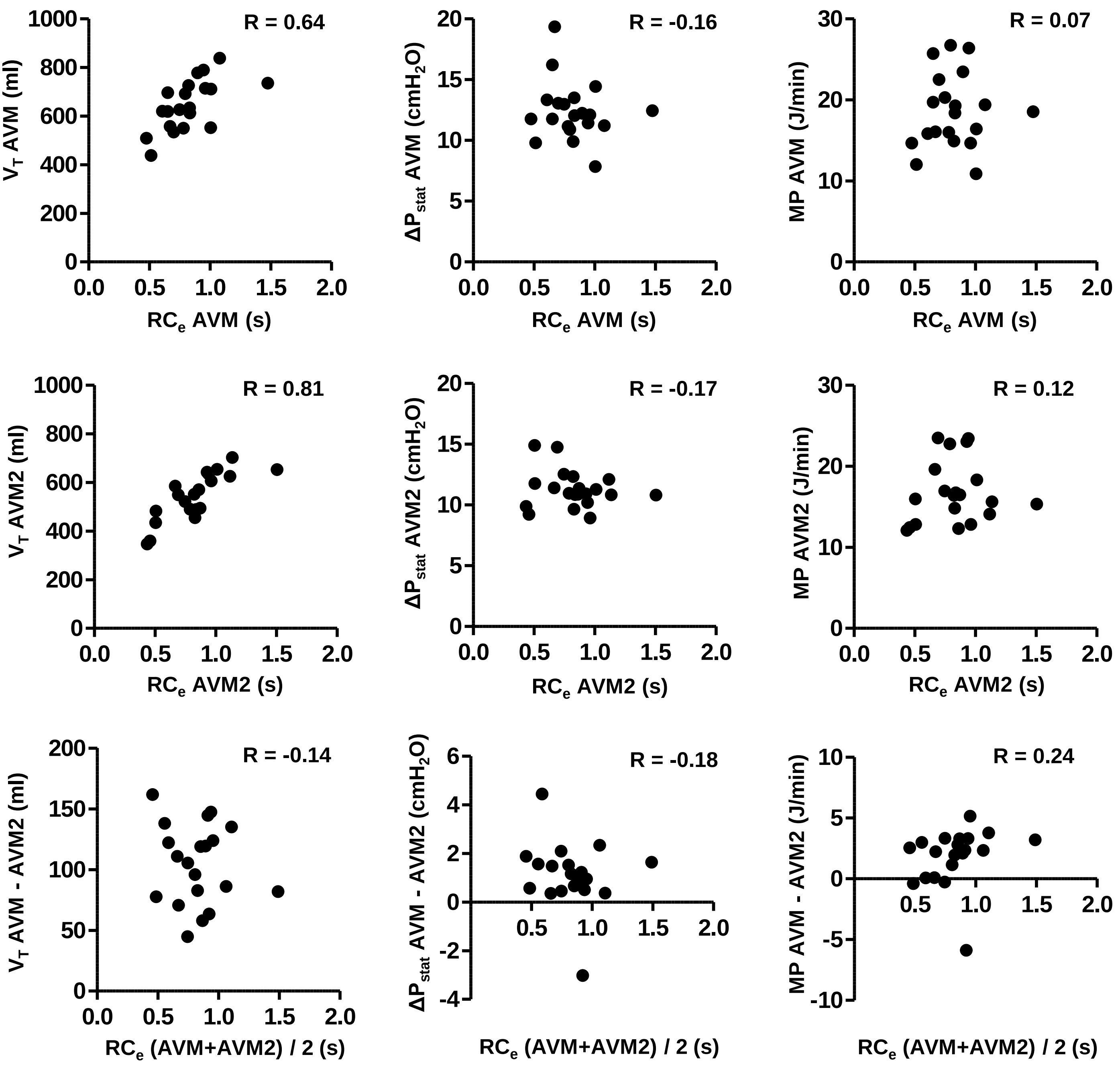


**Figure S1.** Correlation between tidal volume ($V_T$), driving pressure ($\Delta P_{stat}$), mechanical power (MP) and the expiratory time constant ($RC_e$) during adaptive mechanical ventilation with selection of respiratory rate (RR) and $V_T$ according to Otis' equation on least work of breathing (AVM, top row) and with automated minimization of inspiratory power (AVM2, middle row). Bottom row: Difference between AVM and AVM2 for $V_T$, $\Delta P_{stat}$ and MP plotted against mean $Rc_e$ for both modes.
